# Cerebral functional networks during sleep in young and older individuals

**DOI:** 10.1038/s41598-021-84417-0

**Published:** 2021-03-01

**Authors:** Véronique Daneault, Pierre Orban, Nicolas Martin, Christian Dansereau, Jonathan Godbout, Philippe Pouliot, Philip Dickinson, Nadia Gosselin, Gilles Vandewalle, Pierre Maquet, Jean-Marc Lina, Julien Doyon, Pierre Bellec, Julie Carrier

**Affiliations:** 1 Functional Neuroimaging Unit, University of Montreal Geriatric Institute, 4565, Queen-Mary Road, Montreal, QC H3W 1W5 Canada; 2 Center for Advanced Research in Sleep Medicine (CARSM), Hôpital du Sacré-Cœur de Montréal, 5400 Gouin Boulevard West, Montreal, QC H4J 1C5 Canada; 3 Department of Psychology, University of Montreal, Downtown Station, P.O. Box 6128, Montreal, QC H3C 3J7 Canada; 4 Department of Psychiatry, University of Montreal, Downtown Station, P.O. Box 6128, Montreal, QC H3C 3J7 Canada; 5 Génie Électrique, École de technologie supérieure, 1100, rue Notre-Dame Ouest, Montreal, QC H3C 1K3 Canada; 6 École Polytechnique de Montréal, Succursale Centre-Ville, C.P. 6079, Montreal, QC H3C 3A7 Canada; 7 GIGA-Cyclotron Research Centre-In Vivo Imaging, Université de Liège, Allée du 6 Août, Bâtiment B30, Sart Tilman, 4000 Liège, Belgium; 8 Centre de Recherches Mathématiques (CRM), Université de Montréal, Succursale Centre-Ville, Case postale 6128, Montreal, QC H3C 3J7 Canada; 9 Biomedical Engineering Department, McGill University, 3775 University Street, Montreal, QC H3A 2B4 Canada; 10 U678 INSERM, Paris, France; 11 Centre de Recherche de l’Institut Universitaire en Santé Mentale de Montréal, 7331 Hochelaga, Montreal, QC H1N 3V2 Canada

**Keywords:** Circadian rhythms and sleep, Neural ageing

## Abstract

Even though sleep modification is a hallmark of the aging process, age-related changes in functional connectivity using functional Magnetic Resonance Imaging (fMRI) during sleep, remain unknown. Here, we combined electroencephalography and fMRI to examine functional connectivity differences between wakefulness and light sleep stages (N1 and N2 stages) in 16 young (23.1 ± 3.3y; 7 women), and 14 older individuals (59.6 ± 5.7y; 8 women). Results revealed extended, distributed (inter-between) and local (intra-within) decreases in network connectivity during sleep both in young and older individuals. However, compared to the young participants, older individuals showed lower decreases in connectivity or even increases in connectivity between thalamus/basal ganglia and several cerebral regions as well as between frontal regions of various networks. These findings reflect a reduced ability of the older brain to disconnect during sleep that may impede optimal disengagement for loss of responsiveness, enhanced lighter and fragmented sleep, and contribute to age effects on sleep-dependent brain plasticity.

## Introduction

Almost a third of human life is spent sleeping. This fundamental physiological state of reduced consciousness is composed of a series of alternating cycles, non-rapid eye movement (NREM) and rapid eye movement (REM) sleep. NREM state further decomposes into two light sleep stages (N1 and N2) and then to a deeper sleep (N3) stage. Focussing on this descent from wakefulness to NREM sleep in young individuals, recent studies that combined electroencephalography (EEG) and functional magnetic resonance imaging (fMRI) demonstrated a distant and local reorganization of functional networks underlying sensory awareness, information transfer, memory consolidation and executive control^1^. In particular, functional networks showed a breakdown of long-range cortico-cortical functional connectivity together with a stronger local cortical functional integration^2–11^. Specifically, NREM sleep was associated with a lower connectivity between anterior (medial prefrontal cortex) and posterior (posterior cingulate cortex, precuneus) areas of the default-mode network, but strengthened connectivity within posterior nodes^2,12^. Similarly, within the attentional frontoparietal network, NREM sleep, as compared to wakefulness, showed lower long-range connectivity between the intraparietal lobule and frontal areas, but higher connectivity within the intraparietal lobule^9^. Still in NREM sleep as compared to wakefulness, studies reported lower inter-regional connectivity of the sensorimotor network and decreased correlation strength between regions of the executive control network^13^. These results reflect a reduced communication between regions of sensorimotor and central executive networks that would support the fading of sensory awareness and the disengagement of executive control during sleep. The prominent modification in connectivity that occurs with sleep is not restricted to the cortex but also involves subcortical areas including the thalamus and the hippocampus^2,4,8,10^. The general trend toward lower connectivity of distributed (inter/between-networks) versus local (intra/within-network) in the descent to sleep may contribute to an optimal brain-environment disconnection(s) that promotes efficient and stable sleep.

Importantly, as we age, sleep becomes more vulnerable to environmental perturbations^14–16^ and major modifications of NREM sleep are reported (for a review^17^). As compared to younger individuals, older participants show fewer slow waves, fewer sleep spindles^15,18^ and a reduced amount of deep sleep (N3)^17,19^. Older adults almost systematically report that their sleep is lighter and more fragmented^20–22^. There is, unfortunately, a lack of understanding regarding the specific physiological mechanisms that explain these age-related changes in NREM sleep, but recent studies have linked age-related changes in sleep to a global decrease in brain integrity (e.g., cortical thinning, decreased sleep-dependent memory consolidation, increased β-amyloid depositions) and cognitive functions^18,23–25^. The age-related decreases in deep sleep, slow waves and sleep spindles, may reflect a reduced ability to recruit and synchronize local cortical generators of sleep rhythms which drive the crosstalk between cortical areas and favor local over distant connectivity. How distributed and local fMRI connectivity changes during sleep in aging, and whether it may contribute to the age-related deterioration of sleep quality, is not known, however.

Here, we concomitantly recorded the sleep of young and older individuals with EEG and fMRI to investigate the changes in functional connectivity that occur during sleep in healthy aging. We hypothesized that older participants would show a lower breakdown of functional connectivity during NREM sleep as compared to younger ones. In line with this assumption, our results demonstrated that although young and older individuals present similar reductions in functional connectivity when N2 is compared to wakefulness or N1, older individuals also presented significant lower decreases and even increases in distributed (inter-between networks) and local (intra-within network) functional connectivity.

## Results

Sixteen young (23.1 ± 3.3y.o.; 7 women) and 14 older healthy (59.6 ± 5.7y.o.; 8 women) individuals completed the protocol. Given the vulnerability of sleep to disturbances in aging and to increase the likelihood of sleep in the noisy and uncomfortable MR environment, all participants underwent an overnight total sleep deprivation prior to entering the MR scanner after ~ 26 h of continuous wakefulness (see “Materials and methods” section).

### Sleep variables

**Figure 1:**
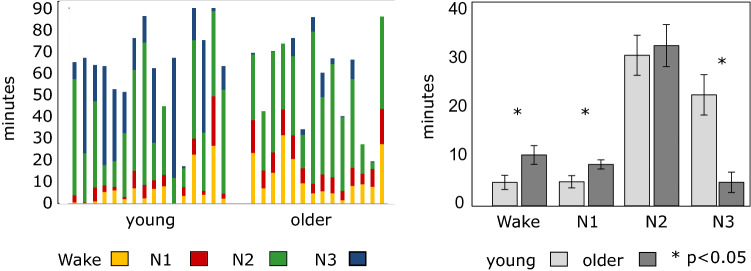
Duration of wakefulness and sleep stages recorded in the scanner. Left panel represents individuals’ times in wakefulness and each sleep stage (duration in min). Right panel represents group values in wakefulness and each sleep stage. Young participants are presented in light grey, older participants in darker grey. Duration of wakefulness, N1, N2, and N3 (mean ± SEM) were recorded in the scanner (after exclusion of EEG epochs associated with high motion in the fMRI series; see “Materials and methods” section).

Figure 1 shows the duration of wakefulness and sleep stages recorded in the MR scanner, after exclusion of EEG epochs associated with high motion in the fMRI series (see “Materials and methods” section), for each individual (left panel) and for the younger and older groups (right panel). Compared to the younger individuals, older participants showed shorter total sleep time (mean ± SD; young: 59 ± 16 min; older: 46.5 ± 19 min; p < 0.05), more time awake (young: 5.9 ± 7.5 min; older: 12.9 ± 9.3 min; p < 0.05), longer duration of N1 (young: 5 ± 5.2 min; older: 8.5 ± 3.7 min; p < 0.05) and less time in N3 (young: 22.9 ± 17.2 min; older: 4.9 ± 8.2 min; p < 0.001). Representative EEG data plots for each age group and each state are displayed in supplementary material (Fig. S1). No significant difference was found for the duration of N2 between the two age groups (young: 31.1 ± 17 min; older: 33.1 ± 16.8 min; p = 0.7). Similar results were observed for raw EEG data (before data exclusion associated with motion) (see Supplementary Table S2). Six older individuals did not reach the N3 stage. Consequently, age-related comparisons involving N3 were not performed.

### Functional connectivity parcellation and significant changes across wakefulness and sleep stages

**Figure 2:**
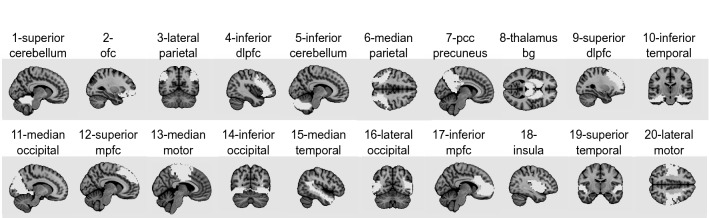
Brain parcellation into 20 clusters. This figure represents each of the 20 functional brain clusters in white on a select brain plane (see “Materials and methods” section). Names and numbers of clusters are as follows: 1—superior cerebellum; 2—orbitofrontal cortex (ofc); 3—lateral parietal cortex; 4—inferior dorsolateral prefrontal cortex (dlpfc); 5—inferior cerebellum; 6—median parietal cortex; 7—posterior cingulate cortex (pcc) and precuneus; 8—thalamus/basal ganglia; 9—superior dorsolateral prefrontal cortex (dlpfc); 10—inferior temporal cortex; 11—median occipital cortex; 12—superior median prefrontal cortex; 13—median motor cortex; 14—inferior occipital cortex; 15—median temporal cortex; 16—lateral occipital cortex; 17—inferior median prefrontal cortex (mpfc); 18—insular cortex; 19—superior temporal cortex; 20—lateral motor cortex.

Using the data driven approach BASC^26^, we parceled the brain into 20 clusters (see Fig. 2). These clusters consistently exhibited similar spontaneous activity across participants, and were spatially stable across both young and older individuals (see “Materials and methods” section on functional brain parcellation).

We then calculated connectivity within and between these 20 brain clusters and we evaluated the percentage of connections showing significant changes between wakefulness, N1 and N2 stages for the young and the older participants, as well as potential interaction with age (see “Materials and methods” section). For the comparisons between wakefulness and N1, no significant connectivity difference in the young and the older groups and no interaction with age were observed. However, a large percentage of brain connections were significantly different between wakefulness and N2 (50% in young, 42% in older individuals) and between N1 and N2 (35% in young; 39% in older individuals). Results provide a correlation value between the matrices of cluster-to-cluster differences for each comparison between stages. Significant interactions with age group, revealing age-related connectivity differences, were also found for the difference between N2 and N1 (16%). As previously stated, the absence of N3 sleep in several older individuals, prevented the evaluation of similar effects and differences between the young and the older groups, for all comparisons with this sleep stage.

### Similar connectivity differences in young and older individuals

To ease the interpretation of our findings, a quantitative mapping of our 20 age-independent clusters was assigned to a more conventional taxonomy of large-scale functional brain networks, the brain networks reported by Yeo^27^. From the parcellation scheme selected, 7 networks are defined: The default-mode network (DMN), the sensorimotor network (SMN), the dorsal attentional network (DAT), the ventral attentional network (VAT), the frontoparietal network (FPN), the limbic network (LIM), and the visual network (VIS). Having no overlap with the cortical atlas of Yeo et al. (2011), 3 clusters from our parcellation were considered as independent: the inferior cerebellum, the superior cerebellum and the thalamus/basal ganglia (see Supplementary Materials, Table S1).

**Figure 3:**
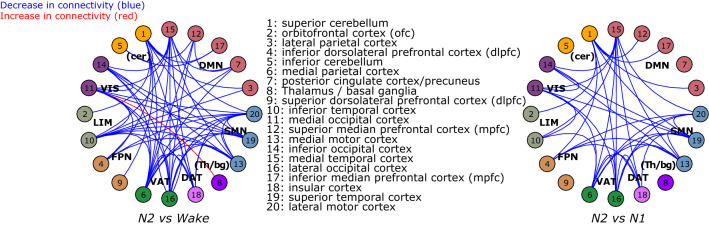
Similar age-group connectivity changes between N2 and wakefulness and between N2 and N1 sleep stage transition. This graph represents changes in functional connectivity between the clusters (two-way directionality) obtained from the data-driven parcellation (circles with numbers) shared by young and older individuals between N2 and wake (left side) and N2 and N1 (right side). Significant connectivity decreases (blue lines) were mostly observed whereas one connectivity increase (red line) was significant between N2 and wake. The seven Yeo’s large-scale networks partitioning the brain are presented with different colors: the default-mode network (DMN); the sensorimotor network (SMN); the dorsal attentional network (DAT); the ventral attentional network (VAT); the frontoparietal network (FPN); the limbic network (LIM) and the visual network (VIS). Clusters of the inferior and superior cerebellum (cer) and the thalamus/basal ganglia (Th/bg) were also added (see Fig. 2 for details on the subdivision of clusters).

Figure 3 shows connectivity changes shared by both age groups between N2 and wakefulness and between N2 and N1, within and between the 20 clusters. It is worth noting that a comparison between N2 and wakefulness revealed large, similar inter-connectivity decreases between the 20 clusters. Similarly, shared connectivity decreases are observed between N2 and N1, but in a more refined manner. Ventral attentional network (VAT) shows significant connectivity decreases between its clusters and with clusters of the dorsal attentional network (DAT) and with several clusters belonging to the sensorimotor (SMN), default-mode (DMN) and visual networks (VIS) both for wakefulness vs. N2 and for N2 vs. N1. Similarly, the sensorimotor network (SMN) shows significant decreases between its clusters and with clusters of the dorsal and ventral attentional (DAT and VAT), visual (VIS) and frontoparietal networks (FPN) for wakefulness vs. N2 and N2 vs. N1 (Fig. 3). In addition, the median temporal cortex of the default-mode network (DMN) shows several significant decreases with remote clusters of the visual (VIS), limbic (LIM), dorsal and ventral attentional (DAT and VAT) and sensorimotor networks (DMN) again for both wakefulness vs. N2 and N2 vs. N1 (Fig. 3).

Representative examples of similar connectivity decrease in young and older individuals (i.e., negative correlation values) for N2 vs. N1 comparisons are illustrated in Supplementary Fig. S2. In addition, a complete illustration of all connectivity changes for N2 vs. wakefulness and N2 vs. N1 in young and older individuals separately are presented in Supplementary Figs. S3–S5.

### Greater decreases in functional connectivity in younger as compared to older individuals

**Figure 4:**
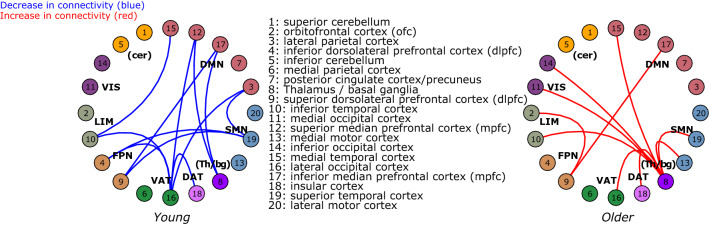
Significant age-group differences in connectivity for the N1 to N2 sleep stage transition. This graph represents significant interaction changes in functional connectivity between the clusters (two-way directionality) obtained from the data driven parcellation (circles with numbers) for the N2 to N1 sleep stage transition. Blue lines (left panel) indicate stronger decreases in connectivity in younger as compared to older individuals. Red lines (right panel) indicate significant connectivity increases in the older groups only. The seven Yeo’s large-scale networks partitioning the brain are presented with different colors: The default-mode network (DMN); the sensorimotor network (SMN); the dorsal attentional network (DAT); the ventral attentional network (VAT); the l frontoparietal network (FPN); the limbic network (LIM) in green and the visual network (VIS). We added the inferior and superior cerebellum (cer) and thalamus/basal ganglia (Th/bg) clusters (see supplementary material for more details on clusters’ subdivision).

**Figure 5:**
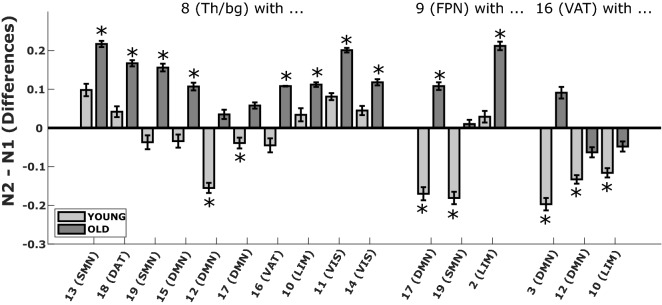
Examples of functional connectivity differences in young and older individuals for N2 vs. N1. This graph is a sample of the connectivity values (R-value; Pearson correlation coefficient) associated with the N2 and N1 comparison between specific clusters and networks that behave differently in young (light grey) and in older individuals (darker grey). Asterisks indicate a significant decrease or increase in functional connectivity for young or/and older groups. Examples of the between-inter networks’ interactions are presented for the default-mode network (DMN), the sensorimotor network (SMN), the dorsal attentional network (DAT), the ventral attentional network (VAT), the frontoparietal network (FPN), the limbic network (LIM) and the visual network (VIS) and thalamus/basal ganglia (Th/bg) clusters (see Fig. 2 and this figure for more details on the subdivision of clusters and for an exhaustive description of functional connectivity differences).

Figure 5 illustrates links for which functional connectivity changes differ between younger and older individuals. Significant interactions with the age group were detected when comparing N2 to N1. As compared to the older individuals, younger participants showed larger connectivity decreases between specific clusters of the ventral attentional network (VAT; lateral occipital cortex), sensorimotor network (SMN; superior temporal cortex), default-mode network (DMN; inferior prefrontal, superior median prefrontal, medial temporal), limbic network (LIM; inferior temporal cortex), frontoparietal network (FPN; dorsolateral prefrontal cortex), and thalamus/basal ganglia (Th/bg) (Fig. 4, left panel).

As illustrated by the right panel of Figs. 4 and 5, compared to the younger participants, older ones show significant increases in connectivity in N2 vs. N1 between the thalamus/basal ganglia (Th/bg) and specific clusters of the sensorimotor network (SMN; superior temporal cortex, median motor), default-mode network (DMN; median temporal cortex), visual network (VIS; median and inferior occipital), limbic network (LIM; inferior temporal cortex) and ventral and dorsal attentional networks (VAT; lateral occipital cortex and DAT; insular cortex). Older individuals also show higher connectivity in N2 vs. N1 between the frontal clusters of the frontoparietal network (FPN; superior dorsolateral prefrontal cortex), the limbic network (LIM; orbitofrontal cortex) and the default-mode network (DMN; median prefrontal cortex). One of the functional links between frontal clusters (frontoparietal network-FPN with default-mode network-DMN) is in fact, not only significantly different between groups, but shows opposite effects in the young (decrease in functional connectivity) and in the older groups (increase in functional connectivity). A complete illustration of all connectivity changes for N2 vs. wake and N2 vs. N1 in young and older individuals separately are presented in Supplementary Figs. S3-S5.

## Discussion

This study first demonstrates that young and older individuals share similar decreases in functional connectivity during N2 as compared to wakefulness and N1. These decreases impact both long-range and local interactions between cerebral regions. Analyses also revealed age-related differences in the change of functional connectivity between N2 and N1, with older individuals showing significantly lower functional connectivity decreases than younger individuals, and even increases in functional connectivity between specific networks.

### Both young and older individuals show decreases in functional connectivity between N2 and wakefulness and between N2 and N1

The fact that we find functional connectivity decreases which are shared by young and older individuals is consistent with previous evidence reporting an overall cortico-cortical functional connectivity breakdown during N2 as compared with wakefulness or N1 in young individuals and over a lifespan^3,5,7,9,10^. The connectivity decreases between sensorimotor, attentional and higher-order cognitive networks^13^, and between all sensory brain areas^28^ were associated with the fading of sensory awareness and the disengagement of executive functions during sleep. Similarly, we also observed decreases in functional connectivity in both age groups between sensory networks (e.g., visual and sensorimotor) and between these networks and networks supporting external-oriented attentional processes (i.e., dorsal and ventral attentional networks). In addition, the sensory networks also showed lower functional connectivity with the frontoparietal network and the default-mode network, associated with cognitive functions and self-relevant thought processes. A generalized disconnection pattern is also observed between the median temporal cluster of the default-mode network associated with memory functions (including the hippocampus and adjacent cortical areas), and all networks (except the frontoparietal network). All these connectivity decreases are consistent with the concept of modularity which refers to a partial segregation and disconnection among brain regions following a progressive reduction of communication and synchrony among distant neural assemblies. Likely induced by massive changes in neuromodulatory tones^29^, the connectivity breakdown during NREM sleep may represent a necessary mechanism to reach optimal efficient fading of sensory awareness/attentional processes and consciousness during sleep in both young and older individuals^3,11,30–32^.

### Older individuals show lower functional connectivity decreases between N2 and N1

Critically, the present study further reveals that young individuals show a larger disconnection between N2 and N1. This decrease in functional connectivity involves specific clusters of the ventral attentional network (lateral occipital cortex), sensorimotor network (superior temporal cortex), default-mode network (inferior prefrontal, superior median prefrontal, medial temporal), limbic network (inferior temporal cortex), frontoparietal network (dorsolateral prefrontal cortex), and thalamus/basal ganglia. Conversely, the lower connectivity decreases in the older brain during NREM sleep suggests a reduced capacity, or a reduced need, to enhance modularity and locally segregated activity during sleep. This result is consistent with resting-state evidence during wake supporting the notion that the older brain is less modular than the younger one^29,33,34^. Specifically, the reduced ability of the older brain to functionally disconnect attentional, sensory and higher cognitive functions**’** related areas, may impede the necessary disengagement for less responsiveness to environmental awareness during sleep^11^. Therefore, lower thalamocortical connectivity with heteromodal cortical areas has been associated with lower consciousness^8^ by preventing cortical excitation transmission and arousal^35^. A lower connectivity decrease between the thalamus/basal ganglia and higher-order frontal cortical clusters of the default-mode networks can induce, in aging, a greater vulnerability of sleep to external disturbances.

### Older individuals show increases in functional connectivity between N2 and N1

While young individuals present decreases in connectivity between frontal clusters of the default-mode and frontoparietal networks, older individuals also show increases in connectivity between frontal clusters of the default-mode network (median prefrontal cortex), frontoparietal network (dorsolateral prefrontal cortex) and the limbic network (orbitofrontal cortex). Frontal disconnections are a major feature of enhanced awareness thresholds^35,36^, and frontal disconnection with parietal areas has been associated with fading of consciousness during propofol-induced anesthesia^36^. This reduced disconnection of frontal areas may therefore be key to the degradation of sleep quality in aging. Interestingly, frontal brain areas are those most associated with age-related decreases in slow waves and sleep spindles^15,37^. Likewise, age-related decreases in SW density and amplitude were related to lower cortical thickness in various cortical areas, including frontal areas^38^. Furthermore, during wakefulness, higher connectivity in older individuals between frontal areas is interpreted as a compensatory mechanism counter-balancing the effects of connectivity changes in other brain networks^39–43^. Our results suggest that a similar process might take place during sleep. It is worth noting that such an increase in connectivity during the descent into deep sleep was also observed using EEG-only connectivity measures with elderly individuals, as compared with younger individuals who rather showed an important disconnection^44,45^.

Lastly, a striking feature of older individuals is an increase in connectivity in N2 as compared to N1 between the thalamus/basal ganglia and clusters of the sensorimotor, default-mode, visual, limbic, dorsal and ventral attentional networks. Interestingly, individuals with insomnia symptoms present altered organization of connectivity networks involving the putamen and the insular cortex of the salience network^46^. Still in those patients, impairments in interhemispheric connectivity between homologues interhemispheric regions are observed, such as hyper arousal of the bilateral thalamus^47^. Higher connectivity between thalamus/basal ganglia and various cortical regions during sleep, might represent a signature of the aging brain, or at least a major feature associated with sleep changes/perturbations/fragilization. One possible interpretation could be that the aging brain manages the challenging environment of the scanner (e.g., its difficulty to disconnect from it) by keeping one network “aware” (e.g., by allowing interactions between the thalamus/basal ganglia with other networks). This brain adaptation might permit modularity increases between other regions, particularly the higher-order ones, to reach the minimal disconnection threshold required to stay asleep. This might help sleep initiation, but at the expense of a lighter sleep.

### Limitations and future directions

The small sample size of our study might have limited the statistical power of our analysis, as well as the nature of significant changes but only for a limited number of contrasts. Since we had strict criteria, the older individuals were very healthy and may not have been representative of the general population. Age-related differences may be even greater in a sample of older individuals who are more representative of the broader population. Furthermore, investigating dynamic connectivity, which revealed specific recurring connectivity patterns for wakefulness and NREM sleep^48^, will help further understanding of sleep connectivity modifications in aging. Other limitations include the greater connectivity instability in wakefulness after sleep deprivation^49^ which possibly reduces the observed differences for comparison including wakefulness. The impact of the current challenging experimental conditions (e.g., sleeping in a scanner) which cannot be considered as ecological sleep, should also be taken into account when considering the present findings. However, reaching N2 sleep is very challenging in an MRI scanner, especially in the older population since their sleep is more vulnerable to disturbances. We selected to study sleep after a sleep deprivation in both age groups to maximize the probability of older individuals falling into a deeper sleep in the scanner. Sleep deprivation may however impact the comparison between young and older individuals. Our work, as well as results from others, have indicated that the effects of sleep deprivation differ in young and older individuals. As compared to young individuals, older participants show a similar or smaller impact of sleep deprivation on waking EEG and cognitive performance and a shallower rebound of slow wave sleep during recovery sleep^16,48–51^. Together, these results suggest that the sleep deprivation may have enhanced age-related differences in functional connectivity between the wakefulness and sleep states. Notwithstanding, we would recommend similar sleep deprivation protocols for future studies which aim to evaluate sleep using fMRI in an older population.

## Conclusion

Although we observed large age-related similarities in connectivity breakdown compatible with fading of awareness and environmental responsiveness, functional reconfiguration of brain networks during NREM sleep stages also differs between young and older individuals. These modifications may underlie age-related differences in the robustness of NREM sleep to withstand external and internal stimulations, as well as age effects on sleep-dependent brain plasticity, which are linked to cerebral integrity and cognitive health in older subjects^17,23,38,50^.

## Materials and methods

### Subjects

Sixteen young adults (8 females; 20–30 years, 23.1 ± 3.3y) and fourteen older individuals (9 females; 52–69 years, 59.6 ± 5.7y) participated in the study. Participants were recruited from the population at large, newspaper advertisements posters on the physical boards of the Research Center and on the University of Montreal campuses, as well as on the electronic bulletin boards of these two institutions. A general interview using standardized questionnaires and homemade questionnaires was used to exclude candidates who reported sleep, medical, neurological or psychiatric disorders. Exclusion criteria also included extreme chronotypes [Morningness-Eveningness Questionnaire-MEQ, scores ≤ 30 or ≥ 70]^51^, excessive daytime sleepiness [Epworth Sleepiness Scale, score > 11]^52^, poor sleep quality [Pittsburg Sleep Quality Index, scores ≥ 7]^53^, high anxiety [Beck Anxiety Inventory, score ≥ 11]^54^ or depression [Beck Depression Inventory-II, scores ≥ 11]^55^ and body mass index > 27 (Table 1). All participants were non-smokers low to moderate consumers of caffeine and alcohol (i.e., ≤ equivalent of 600 mg of caffeine units/day; ≤ equivalent of 1250 ml of wine/week) and right-handed (Table 1). None of the participants was using medication known to affect the central nervous system, had worked on night shifts during the past year or had travelled through more than one-time zone during the past 3 months. All participants underwent urine and blood tests as well as a standard polysomnography to rule out significant medical conditions including sleep apnea (exclusion criteria: index per hour > 10) and periodic leg movement (exclusion criteria: index per hour > 10 associated with arousals) syndrome. Young and older participants did not differ on body mass index, anxiety, depression scores or sleep quality (Table 1). As expected^20–22^, older participants showed higher morningness scores, earlier bedtimes and wake times, and shorter habitual sleep duration compared to the young adults (Table 1). The study protocol was approved by the research ethics board of the *Regroupement Neuroimagerie Québec* (*RNQ*) and all experiments were performed in accordance with relevant guidelines and regulations. All participants gave their written informed consent and received a financial compensation.

**Table 1:** Subject characteristics (mean ± SD).

	Older subjects (> 50y; n = 14)	Younger subjects (20–30y; n = 16)	*p* value
Age	59.6 ± 5.7	23.1 ± 3.3	–
Laterality (right-handed)	14	16	
Sex	6M/8F	9M/7F	0.46
Body mass index (BMI)	24.9 ± 4.3	23.3 ± 3.4	0.29
Depression score (BDI-II)	2.1 ± 3.2	2.4 ± 2.6	0.73
Anxiety score (BAI)	0.9 ± 0.6	2.2 ± 3.3	0.10
Daytime propensity to fall asleep (Epworth)	6.7 ± 3.9	5.6 ± 3.9	0.42
Sleep disturbance score (PSQI)	2.5 ± 1.9	2.8 ± 1.7	0.64
Chronotype score (MEQ)	65.8 ± 6	48.3 ± 7.6	0.52
Years of education	14.9 ± 3	15.8 ± 2.4	0.36
Coffee/Tea intake per week (cup)	12.1 ± 8.3	7.5 ± 6.7	0.15
Alcohol intake per week (glass)	2.5 ± 2.2	2.3 ± 2.3	0.75
Ratio of women using hormonal contraceptive	0/8	5/7	< 0.001*
Ratio of women using hormonal replacement therapy	1/8	0/7	0.25
Date of study (dd/mm/yy)	08/11/09 ± 28d	07/11/09 ± 29d	0.88
**Bedtime prior to experiment**			
PSQI-week	22:34 ± 0:55	24:02 ± 0:33	< 0.001*
Sleep diary	22:36 ± 0:44	24:03 ± 0:41	< 0.001*
**Wake time prior to experiment**			
PSQI-week	6:49 ± 0:43	8:40 ± 0:59	< 0.001*
Sleep diary	6:55 ± 0:59	8:31 ± 0:59	< 0.001*
Total sleep time prior to experiment (h:min) (PSQI-week)	6:49 ± 0:43	8:12 ± 0:48	< 0.002*

*BDI-II* beck depression inventory-II, *BAI* beck anxiety inventory, *PSQI* Pittsburgh sleep quality index, *MEQ* morningness–eveningness questionnaire.

### Research protocol

Seven days prior to data acquisition, subjects had to follow a regular sleep–wake schedule based on their habitual bed and wake times (± 30 min), as monitored by wrist actigraphy and daily sleep diaries. They were instructed to avoid naps, intense physical activity, and caffeine- or alcohol-containing beverages on admission day. Sleep recordings in the fMRI scanner were acquired in the morning after 26 h of sleep deprivation, i.e., in conditions of enhanced age effects on slow wave sleep^56,57^. Subjects arrived at the laboratory 12 h after their habitual wake time (early evening) and were kept awake under technician supervision, in dim light (˂ 15 lx) for 14 h (throughout the night). Two hours after their habitual wake time (26 h of wakefulness), participants were placed in the scanner, the lights of the MRI scanning room were closed and the participants were instructed “to relax, to close their eyes and to let sleep come”. Simultaneous EEG-fMRI were recorded for a maximum period of 100 min.

### EEG/fMRI acquisition

Simultaneously with fMRI acquisitions, we recorded scalp-surface EEG using a 64-electrode cap (BrainCap MR; Brain Products; referenced to FCz) and electrocardiography using 3 bipolar electrodes (Brain Products). Electrode–skin impedance was kept below 5 kΩ (plus 10 kΩ built-in resistors) using abrasive paste (Nuprep; Weaver and Company) and electrode gel (Electro-gel; Electro-cap International) to ensure stable data acquisition throughout the recording session. EEG data were recorded with two 32-channel amplifiers (BrainAmp MR plus, Brain Products) and ECG data with one 16-channel amplifier (BrainAmp ExG MR, Brain Products). Data were analog-filtered (0.016–250 Hz) and digitized (5 kHz sampling rate; 500 nV resolution) with BrainVision Recorder 1.20 (Brain Products).

fMRI time series were acquired in a 3.0-T scanner (Magnetom TIM Trio, Siemens) with an echo planar imaging sequence (voxel size = 3.4 × 3.4 × 3.0 mm; 32 transversal slices; [TR] = 2460 ms; [TE] = 40 ms; flip angle = 90°; [FOV] = 220 mm). A structural T1-weighted image was also acquired with a MP-RAGE sequence (voxel size = 1.0 × 1.0 × 1.0 mm; 176 sagittal slices; TR = 2300 ms; TE = 2.91 ms; flip angle = 9°; FOV = 256 mm).

### EEG analysis: sleep stage scoring

Scanner gradient artifacts were removed from EEG recordings by adaptive subtraction with Brain Vision Analyzer (Brain Products)^58^. Any residual gradient artifacts were further removed using independent component analysis (ICA). A Ballistocardiographic artifact was filtered out (FAST toolbox)^59^, and movement artifacts were numerically detected^60^. EEG data were down-sampled to 250 Hz, low-pass filtered with an infinite impulse response filter (− 48 dB at 0.3–50 Hz), and re-referenced from FCz to averaged mastoids (i.e., TP9 and TP10). Sleep stages were visually scored by an experienced technician in 20-s epochs according to standard criteria^61^ using commercial software (Harmonie, Stellate System). SWs were detected automatically on artifact-free NREM in Cz and Fz scalp derivations. Data were initially filtered between 0.3 and 4.0 Hz using a band-pass filter (− 3 dB at 0.3 and 4.0 Hz; − 23 dB at 0.1 and 4.2 Hz). SWs were detected on NREM sleep using the following previously published criteria: (1) negative peak <  − 40 μV, (2) peak-to-peak amplitude > 75 μV, (3) duration of negative deflection > 125 ms and < 1500 ms, and (4) duration of positive deflection < 1000 ms^62^. For Cz and Fz derivations, SW density was defined as the number of SWs per minute of N2 and N3 sleep stages. SW densities were averaged over N2 and N3 sleep stages.

### fMRI analysis and computational environment

fMRI data were processed using the NeuroImaging Analysis Kit version 0.12.18 (NIAK), under CentOS with Octave (http://gnu.octave.org) version 3.6.1 and the Minc toolkit (http://bic-mni.github.io/) version 0.3.18. Analyses were executed in a high-performance computing environment (http://www.hpc.mcgill.ca/http://www.hpc.mcgill.ca/), using the pipeline system for Octave and Matlab (PSOM)^63^. A detailed description of the pipeline can be found on the NIAK website (http://niak.simexp-lab.org/build/html/index.html).

### fMRI data preprocessing: registration

Each time series was corrected for inter-slice difference**s** in acquisition time and rigid body motion (within and between runs). For each subject, the median motion-corrected volume was co-registered with a T1 individual scan using Minctracc^64^, which was itself nonlinearly transformed to the Montreal Neurological Institute (MNI) nonlinear template^65^ using the CIVET pipeline^66^. The rigid-body, fMRI-to-T1 and T1-to-stereotaxic transformations were all combined to resample the fMRI in MNI space at a 3 mm isotropic resolution.

### Quality control: registration

Each scan for each run was individually reviewed to assess for anatomical/functional alignment according to visual evaluation with an early iteration of the protocol described in Benhajali et al.^67^. For cases that failed the quality control, the structural and functional volumes were manually centered, prior to automated registration. All subjects passed quality control after these adjustments.

### Quality control: motion

It has been shown that participant motion can have large effects on functional connectivity estimates, especially for older adults^68^. Volumes showing a frame displacement greater than 0.5 mm were removed through a censoring method, which has been shown to contribute to spurious connectivity^69,70^. The overall number of volumes left after censoring did not differ significantly between age groups (young: 1625 ± 462; older: 1501 ± 536; p = 0.5). However, the amount of residual motion for the volumes retained after censoring (residual frame displacement) was significantly higher in older than in younger subjects (young: 0.15 ± 0.04; older: 0.21 ± 0.06; p < 0.001). For this reason, average frame displacement measure per subject was included as a confounding variable in group-level statistical tests. Note that there was no significant interaction between the residual frame displacement of distinct stages and age group (p > 0.1). Also, time series with less than 2 min of acquisition lead to estimates of functional connectivity with poor test–retest reliability^71^. A minimum number of 60 volumes surviving censoring, corresponding to 180 s of acquisition, were thus required for further analysis of each of the stages (wakefulness, N1, N2 and N3). For this reason, some subjects were rejected from the analyses of specific stages: 2 young subjects for wakefulness, 2 young subjects for N1, as well as 1 young and 8 older subjects for N3. Note that longer time series would have resulted in a better reliability, but would have further decreased the sample size.

### Confound regression and smoothing

To reduce spurious correlations arising from other various noise sources^72^, the following nuisance covariates were regressed out from fMRI time series: slow time drifts (basis of the discrete cosines with a 0.01 Hz high-pass cut-off), average signals in conservative masks of the white matter and the lateral ventricles (in order to control for nuisance factors—cardiac and respiratory noise) as well as the first principal components (accounting for 95% variance) of the six rigid-body motion parameters and their squares. The fMRI volumes were finally spatially smoothed with a 6 mm isotropic Gaussian blurring kernel.

### Functional brain parcellation

In order to characterize the effects of different states on functional connectivity for brain networks whose topological characteristics may differ in older individuals, we derived a unique set of brain parcellations based on our mixed sample of young and older individuals, with a spatial resolution of 20 clusters. To this end, a bootstrap analysis of stable clusters (BASC)^26^ generated sets of functional brain clusters that consistently exhibited similar spontaneous activity in individual subjects, and that were also spatially stable across both young and older participants. Briefly, this data-driven algorithm first consists of a cluster-growing algorithm to reduce data dimension to a time × space array with about 1000 functional clusters. BASC then replicates a hierarchical Ward clustering on 1000 bootstrap data samples and estimates the probability that a pair of clusters falls in the same cluster, a measure called stability. The cluster × cluster stability matrix is fed into a clustering procedure to derive consensus clusters, which are composed of clusters with a high average probability of being assigned to the same cluster across all replications. This general principle is applied first at the individual level (i.e., bootstrapping time series), and then at the group level (i.e., bootstrapping subjects). BASC is applied using the whole fMRI runs (i.e., encompassing both wakefulness and sleep stages) and all subjects, i.e., both young and older subjects, as to provide a single and unbiased set of 20 clusters to compare connectivity values across sleep stages and age groups.

### Connectome-wide association analysis

Mass univariate parametric regression tests were conducted on Pearson correlation coefficients used as indices of functional brain connectivity, as widely adopted in fMRI connectivity sleep studies (e.g.^73^). For every participant and pair of clusters at a given resolution, between-clusters connectivity was measured by the Fisher transform of the Pearson’s correlation between the average (over clusters) time series of a pair of clusters (two-way directionality). The within-cluster connectivity of a given cluster was the Fisher transform of the average correlation between the time series of all pairs of the clusters within that cluster. For each participant, epochs corresponding to each state based on concurrent EEG scorings were extracted from the entire functional run, and average measures of connectivity across epochs were derived independently for each state (wake, N1, N2, N3). For every distributed (inter-between) and local (intra-within) network connectivity measure, a separate group-level general linear model (GLM) was implemented to test for differences in connectivity across states, in each age group separately. For instance, when N2 is compared to wakefulness (resp. N1), connectivity changes were consistent across age groups with a correlation between the matrices of 0.77 (resp. 0.66), with significant connectivity decreases replicated in young and older participants (similarity coefficient value DICE index: 0.67 and 0.54).

### Number of brain clusters and multiple comparisons

Given K functional brain clusters generated by BASC, there are K(K − 1)/2 inter-cluster connectivity measures, and K within-cluster measures, for a total of K(K + 1)/2 distinct regression tests being performed. A Benjamini–Hochberg false discovery rate (FDR) was implemented to correct for multiple comparisons^74^. A relatively liberal threshold qFDR < 0.1 was selected, to limit the number of false negatives. The validity of this approach to multiple comparisons in combination with parametric regression tests was evaluated on both simulations and real data^75^. The evaluation resulted in a tight control of the effective FDR, yet also stressed a loss in sensitivity with increasing K. The evaluation recommended to use a K in the range 30–50, however due to our limited sample size, we selected K = 20 for our primary analysis.

### Brain clusters/parcels and classical brain network correspondences

In order to provide a graphical interpretation of our results, a quantitative mapping of the 20 brain clusters/parcels was performed onto the Yeo functional atlas^27^ of 7-brain subdivisions. This correspondence was done by estimating the overlap between the actual data-driven parcellation and the following canonical networks: Default-Mode Network (DMN), Dorsal-Attentional Network (DAT), Ventral-Attentional Network (VAT), Limbic (LIM), Sensorimotor Network (SMN), Visual (VIS) and Frontoparietal Network (FPN). The correspondence and overlap values between the 20 clusters and the Yeo’s networks are given in Table S1 (see the top panel of Figs. S2, S3 for the representation of our 20 functional brain clusters).

## Supplementary Information

Supplementary Information.

## Data Availability

The datasets generated during and/or analysed during the current study are available from the corresponding author on reasonable request.
