# Supplementary material for: Cerebral functional networks during sleep in young and older individuals
Source: Sci Rep. 2021 Mar 1;11:4905. doi: 10.1038/s41598-021-84417-0 (PMC7921592; doi:10.1038/s41598-021-84417-0)
Supplement: Supplementary file 1 — Supplementary Information. [file 41598_2021_84417_MOESM1_ESM.pdf]

Cerebral functional networks during sleep in young and older individuals

Véronique Daneault, Pierre Orban, Nicolas Martin, Christian Dansereau, Jonathan Godbout, Philippe Pouliot, Philip Dickinson, Nadia Gosselin, Gilles Vandewalle, Pierre Maquet, Jean-Marc Lina, Julien Doyon, Pierre Bellec, Julie Carrier

SUPPLEMENTARY TABLES AND FIGURES

TABLE

TABLE S1: Correspondence between the 20 clusters and the Yeo's defined networks

Our Cluster	Overlap (%)	Absolute overlap (Voxels)	Overlap of local cluster (%)	Overlap of yeo region (%)	Yeo Number and Region Name
1-Superior cerebellum	0.01	927	0.94	0.01	1-Visual
2-Orbitofrontal cortex	0.42	23559	0.55	0.34	5-Limbic
3-Lateral parietal cortex	0.15	18983	0.75	0.08	7-Default mode
4-Inferior dorsolateral prefrontal cortex	0.25	25496	0.43	0.17	6-Fronto-parietal
5-Inferior cerebellum	0.02	1353	0.89	0.01	1-Visual
6-Medial parietal cortex	0.34	28288	0.56	0.24	3-Ventral Attention
7-Posterio cingular cortex/precuneus	0.19	26858	0.49	0.12	7-Default mode
8-Thalamus-Basal Ganglia	0.01	407	0.62	0.00	4-Dorsal Attention
9-Superior dorsolateral prefrontal cortex	0.41	44094	0.66	0.30	6-Fronto-parietal
10-Inferior temporal cortex	0.52	31083	0.62	0.44	5-Limbic
11-Medial occipital cortex	0.54	66924	0.91	0.38	1-Visual
12-Superior medial prefrontal cortex	0.26	35726	0.78	0.15	7-Default mode
13-Medial motor cortex	0.41	46504	0.62	0.31	2-Somatomotor
14-Inferior occipital cortex	0.61	79208	0.94	0.45	1-Visual
15-medial temporal cortex	0.31	44406	0.77	0.19	7-Default mode
16-Lateral occipital cortex	0.26	21122	0.50	0.18	3-Ventral Attention
17-Inferior median prefrontal cortex	0.24	34278	0.69	0.15	7-Default mode
18-Insular cortex	0.30	24822	0.42	0.23	4-Dorsal Attention
19-Superior temporal cortex	0.30	31942	0.51	0.21	2-Somatomotor
20-Lateral motor cortex	0.31	38886	0.40	0.26	2-Somatomotor

This table represents an overlap between our 20 defined-clusters and the Yeo et al. 2011 parcellation. The first row indicates the name and number of our clusters; the second row refers to the overlap percentage of our cluster with the best matching Yeo region; the third row is the absolute overlap of the two clusters/regions in voxels (note that our atlas was resampled to match the 1mm isotropic voxels of the Yeo parcellation so there are many more voxels); the fourth row represents the percentage of our cluster that overlaps with the Yeo region (note that 100% means that 100% of our cluster is inside the union mask); the fifth row is the percentage of the corresponding Yeo region that overlaps with our cluster (same constraints apply as for the fourth row); and the sixth row refers to the number and name of the Yeo region.

TABLE S2: Polysomnography parameters from the EEG recordings

	Young (mean±sem)	Older (mean±sem)	P
EEG total recording time	70 ± 3.4	76.3 ± 2.8	0.2
Wake	7.5 ± 1.9	16.1 ± 2.6	0.01
N1	5.3 ± 1.3	10.7 ± 0.9	0.002
N2	32.9 ± 4.1	43.7 ± 3.9	0.07
N3	24.3 ± 4	5.8 ± 2.1	<0.001
Total NREM duration	62.5 ± 2.9	60.2 ± 3	0.6
REM duration	--	--	--
Total number of awakenings	7.9 ± 2.6	16.3 ± 1.8	0.01
SW density at CZ (N2)	6.75 ± 0.86	2.7 ± 0.46	<0.001
Total SW density at CZ (N2+N3)	16.25 ± 2.48	4 ± 0.82	<0.001
SW density at FZ (N2)	10 ± 1.07	3.65 ± 0.47	<0.001
Total SW density at FZ (N2+N3)	22.71 ± 3.11	5.58 ± 1.32	<0.001

This table represents polysomnography parameters obtained from the EEG recordings before any data exclusion (no scrubbing related to high motion in the scanner). Please note that no REM was recorded for any of the participants as the recording lasted a maximum period of 100 minutes. In addition, we did not report any value related with spindles because an MRI artifact in the frequency range of these neurophysiological sleep correlates prevented a reliable detection. Acronyms: Electroencephalography (EEG), Non-Rapid-Eye-Movement (NREM), Rapid-Eye-Movement (REM), Slow Wave (SW), Derivations Cz (central electrode) and Fz (frontal electrode in mid-sagittal plane).

T-tests results indicated a significant effect of age group for minutes of wake, N1 and N3 and a tendency for older individuals to spend more time in N2 as compared to young individuals. As compared to the younger ones, older individuals also showed more awakenings and a lower SW density for Cz and Fz derivations.

FIGURES

FIGURE S1

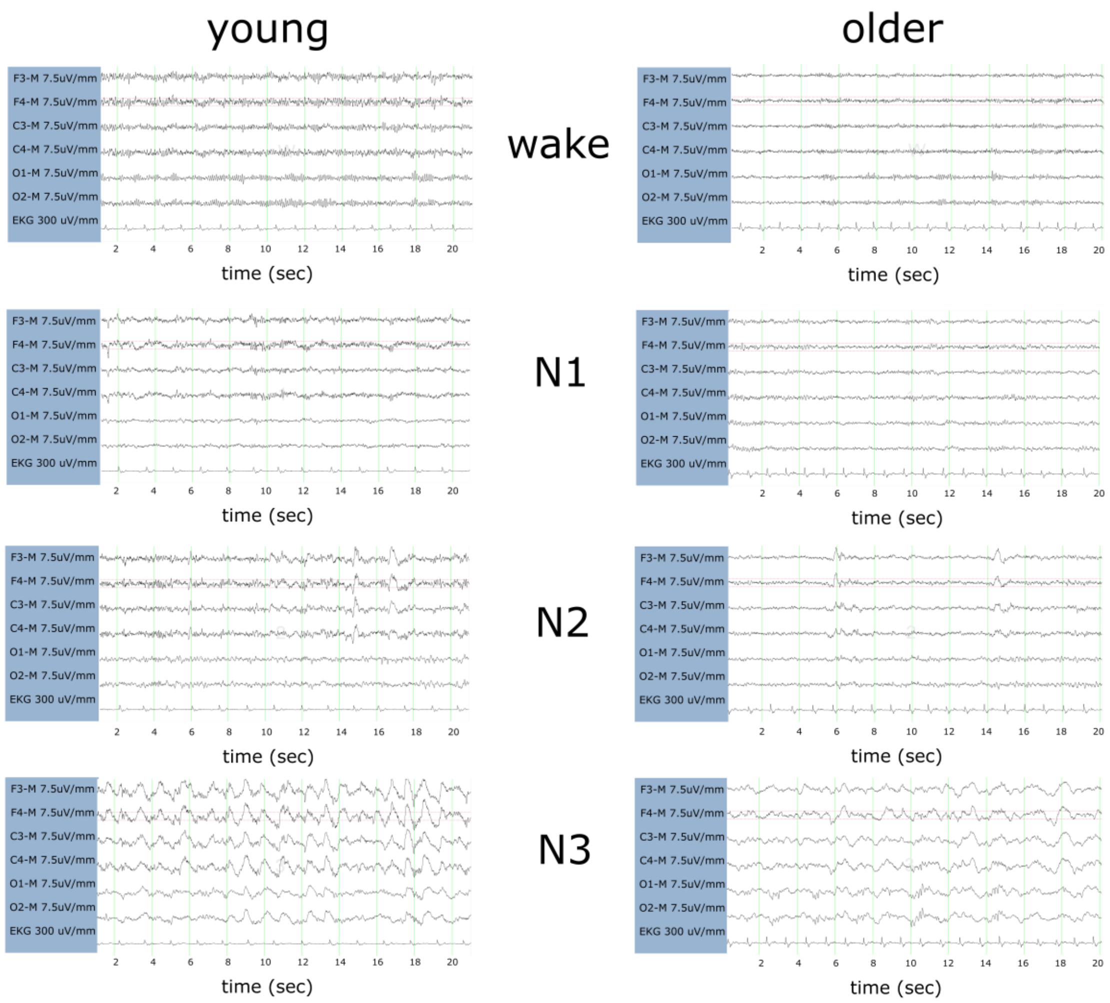

FIGURE S1: Representative EEG data after artifacts removal for each age group and each state.

This figure represents examples of EEG plots (1 epoch of 20 seconds) recorded in the MRI scanner after artifacts removal for wake, N1, N2 and N3 for one young individual (left side) and for one older individual (right side). Six electrodes referenced to the mastoid (M) are presented (e.g., F3-M, F4-M, C3-M, C4-M, O1-M, O2-M), as well as is the EKG which is related to the electrocardiogram derivation. A horizontal scale represents time in seconds and the vertical scale illustrates the electrodes. The sensitivity of the horizontal scale was set at 7.5uV/mm for the brain electrodes and at 300uV/mm for the EKG. Please note that the interval between the dotted pink lines shown at F4-M indicates an amplitude of 75uV.

FIGURE S2

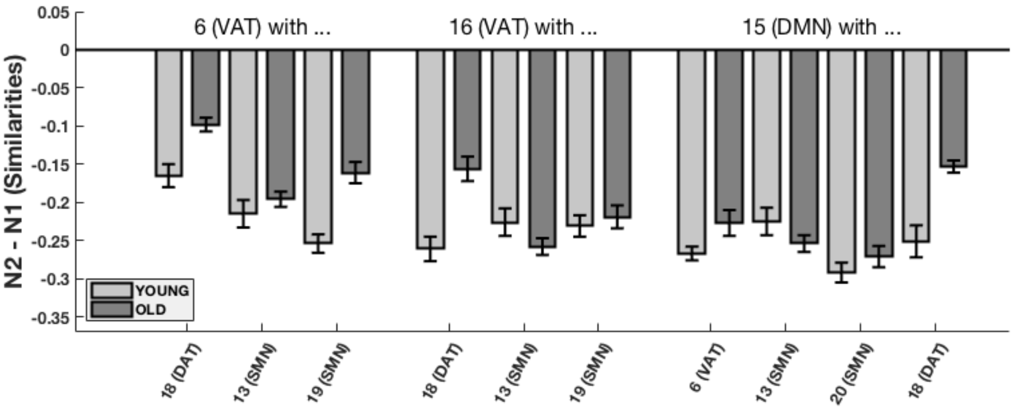

FIGURE S2: Representative examples of similar functional connectivity differences in young and older individuals for N2 vs. N1.

This graph is a sample of the connectivity values (R-values; Pearson correlation coefficient) associated with the N2 and N1 comparison. Significant similar decreases in functional connectivity after correction (FDR-corrected) in young (light grey) and older individuals (darker grey) are shown. Examples of between-inter networks interactions are presented for the default-mode network (DMN), the sensorimotor network (SMN), the dorsal attentional network (DAT) and the ventral attentional network (VAT) (see Figure 2 and Figure S3 for more details on clusters' subdivision and an exhaustive description of the similar functional connectivity).

Representative examples of similar connectivity decrease in young and older individuals (i.e., negative correlation values) for N2 vs. N1 comparisons are illustrated in Figure S2. In each age group we observed that clusters 6 (medial parietal cortex) and 16 (lateral occipital cortex) of the ventral attentional network (VAT; Figure S2 left and central panel) showed significant decreases with specific clusters of the dorsal attentional (DAT) and the sensorimotor networks (SMN). Clusters 15 (median temporal cortex) of the default-mode network (DMN) also shows significant decreases in functional connectivity with specific clusters of VAT and SMM (Figure S2 left panel).

FIGURE S3

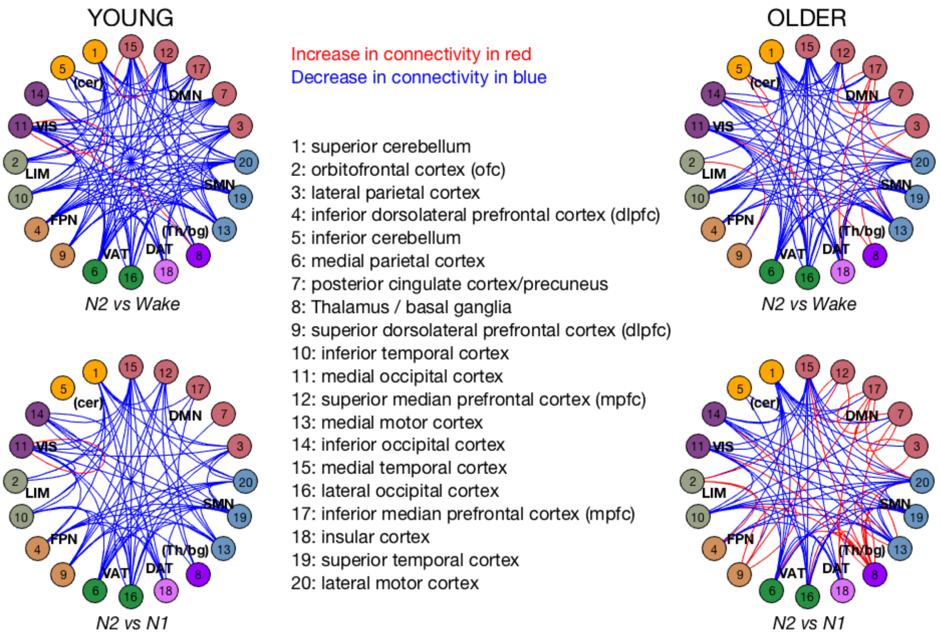

FIGURE S3: Exhaustive representation of connectivity changes in each age group (young and older individuals) for each investigated states' comparison (N2 vs. wake and N2 vs. N1).

This graph represents changes in functional connectivity between the clusters (two-way directionality) obtained from the data driven parcellation (circles with numbers) for young and older individuals separately and between N2 and wake (left side) and N2 and N1 (right side). Significant connectivity decreases (blue lines) were mostly observed whereas one connectivity increase (red line) was significant between N2 and wake. The seven Yeo's large-scale networks partitioning the brain are presented with different colors: the default-mode network (DMN); the sensorimotor network (SMN); the dorsal attentional network (DAT); the ventral attentional network (VAT); the frontoparietal network (FPN); the limbic network (LIM) and the visual network (VIS). Clusters of the inferior and superior cerebellum (cer) and the thalamus/basal ganglia (Th/bg) were also added (see Figure 2 for details on the subdivision of clusters).

FIGURE S4

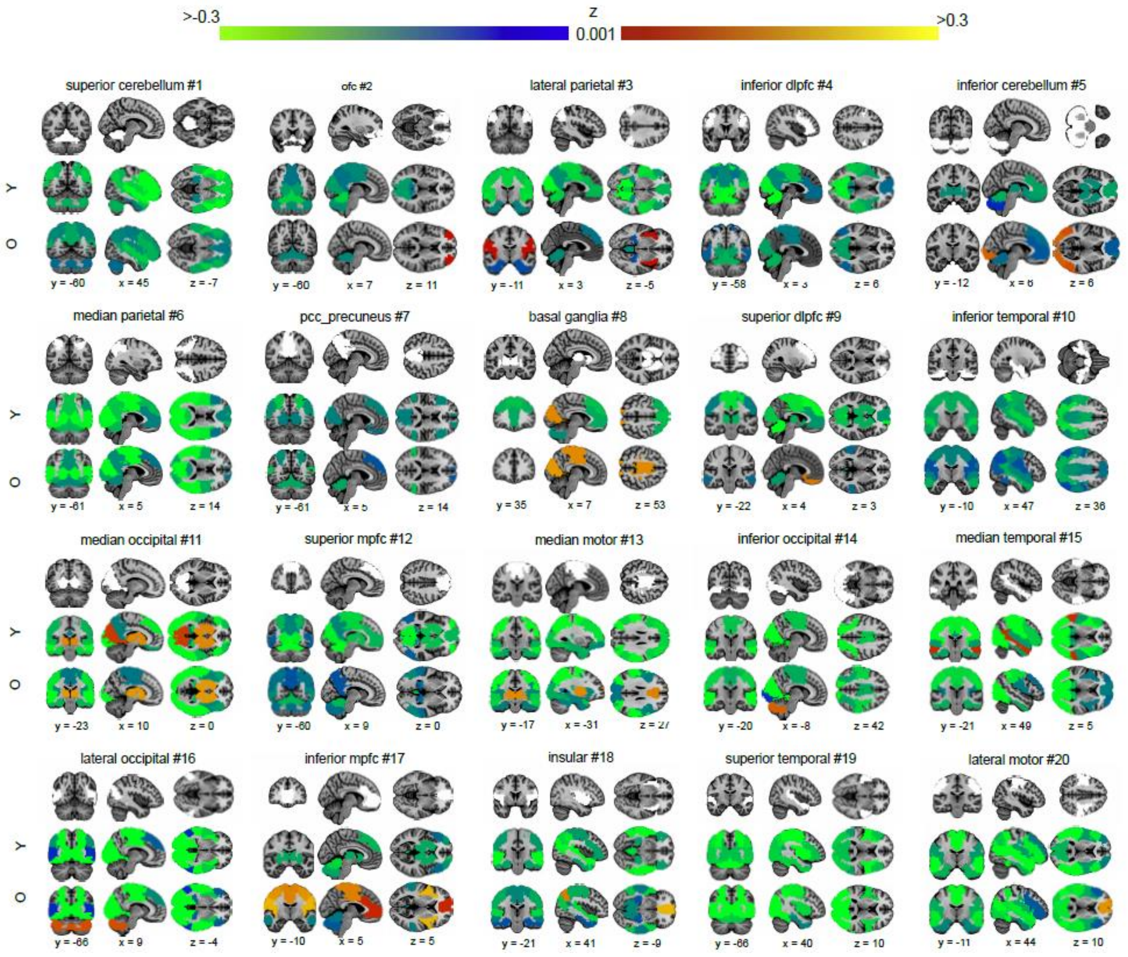

FIGURE S4: Complete connectivity maps for N2 versus wake

Top panel represents each of the 20 functional brain clusters. Each subsequent row depicts the change in connectivity for each group separately (second and third row). A color bar indicates decreased (cold colors) or increased (hot colors) in FC.

FIGURE S5

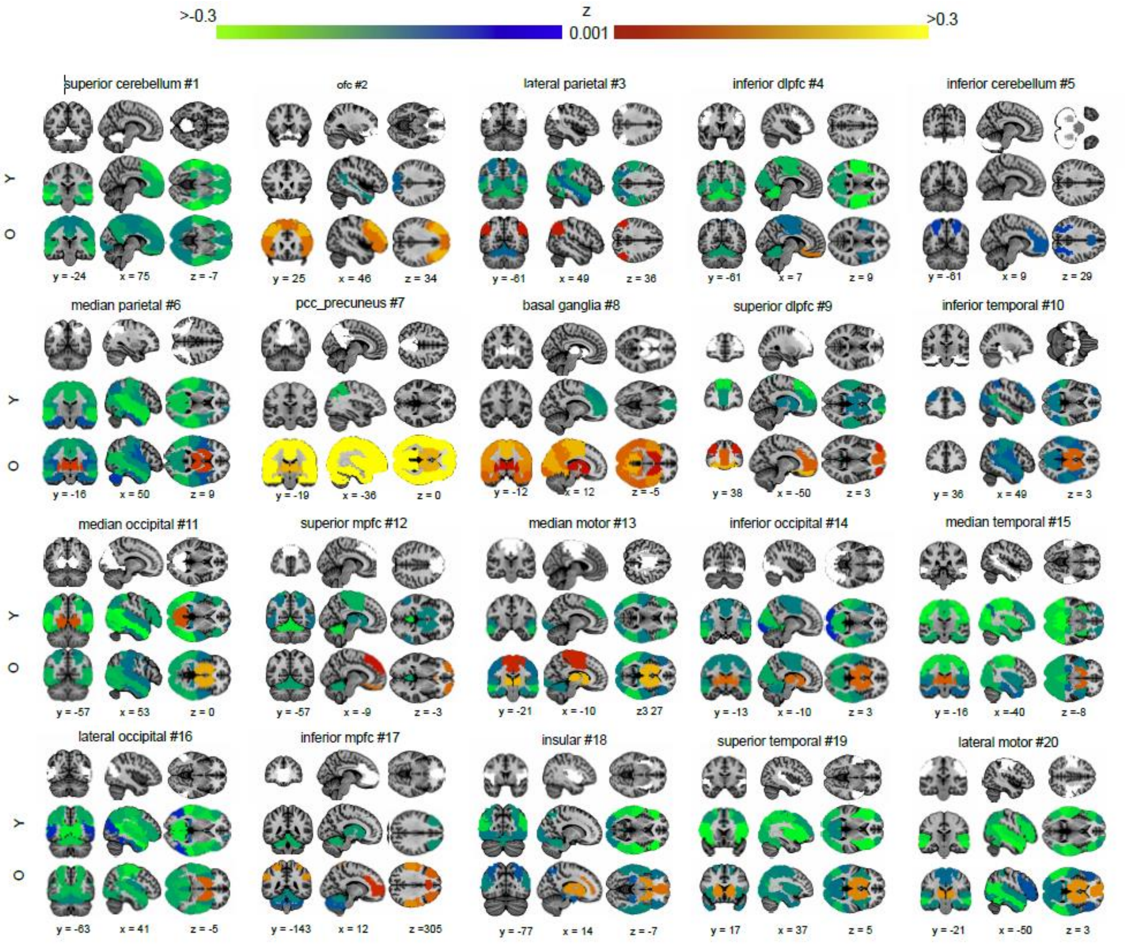

FIGURE S5: Complete connectivity maps for N2 versus N1

Top panel represents each of the 20 functional brain clusters. Each subsequent row depicts the change in connectivity for each group separately (second and third row). A color bar indicates decreased (cold colors) or increased (hot colors) in FC.
